# Multi-omics reveals novel forage advantages of *Potentilla anserina* Linnaeus in high-salt habitats

**DOI:** 10.3389/fmicb.2025.1659904

**Published:** 2025-12-03

**Authors:** Zhijia Cui, Xiaoling Zhang, Ziyang Lv, Shangkun Yang, Miaomiao Zhang, Hanghang Hou, Jing Li, Yuhao Yuan, Junqiao Li, Baili Feng

**Affiliations:** 1 State Key Laboratory of Crop Stress Biology for Arid Areas, College of Agronomy, Northwest A&F University, Xianyang, Shaanxi, China; 2 Laboratory of Innovation and Utilization of Crop Germplasm Resources in Qinghai Province, and Key Laboratory of High-Value Utilization of Specialty Economic Plants, College of Ecology, Environment and Resources, Qinghai Minzu University, Xining, China; 3 School of Life Sciences/Shaanxi Key Laboratory of Research and Utilization of Resource Plants on the Loess Plateau, Yan‘an University, Yan'an, Shaanxi, China; 4 College of Agronomy, Henan Agricultural University, Zhengzhou, Henan, China

**Keywords:** *Potentilla anserina* Linnaeus, volatile metabolites, non-volatile metabolites, bacteria, fungus

## Abstract

**Introduction:**

*Potentilla anserina* Linnaeus (*P. anserina*) is a traditional Chinese herbal medicine with ethnic characteristics that grows in the Qinghai-Tibetan Plateau. It has the potential to be used as a novel feed for ruminants. However, the large area of saline-alkaline soils makes it difficult to rationally use Portulaca oleracea as a feed.

**Methods:**

In this study, the effects of volatile metabolites, non-volatile, bacteria and fungi in stems and leaves of *P. anserina* under three different treatments (fresh grass, hay and silage) in high-salt were investigated using metabolomics and microbiological methods.

**Results:**

Silage under salt stress also improved crude protein and crude fat content compared to hay and fresh treatments. A total of 996 volatile and 928 non-volatile metabolites were identified. Among them, the main volatile substance of silage was 1-Nonen-3-one, while the non-volatile substance was 3-O-Methylgalangin. SC-I-84, Methyloversatilis, and Pseudomonas was specific to *P. anserina* forage, while Podosphaera is greatly reduced in high-salts. The Pseudomonas bacteria produced specifically improved the drought resistance and salt tolerance of *P. anserina*.

**Discussion:**

These findings provide essential insights for valorizing *P. anserina* as a sustainable feed resource, supporting its potential application in animal production within saline-alkaline environments.

## 1 Introduction

As the issue of global food security has become more prominent, the efficient development and sustainable use of land resources has become a focus of attention. However, the large amount of saline land is often seen as a resource that is difficult to exploit due to its low yield and high conversion costs, but in reality saline land has great potential as a reserve of arable land (Negacz et al., 2022; Du et al., 2023). Plants are grown to improve soil physical and chemical properties, soil enzyme activity and soil micro-organisms. Thus, by applying scientific improvement techniques and rational land use planning, high-salts can not only provide new space for food production, but also play a positive role in ecological protection and environmental management (El Shaer, 2010).

In recent years, with the rapid development of the livestock industry, the demand for high quality pasture resources has increased dramatically, but the available pasture land has become increasingly scarce, leading to a growing contradiction between pasture supply and demand (Keeling et al., 2019). In this context, saline and alkaline soils have gradually emerged as a potential breakthrough in solving the problem of pasture resource scarcity due to their wide distribution and underutilized characteristics (El Shaer, 2010). At present, research on the cultivation of salt-tolerant fodder grasses in saline and alkaline lands at home and abroad has made some progress, such as the selection and breeding of salt-tolerant fodder grass varieties, the optimisation of soil improvement techniques, and the construction of highly efficient and low-cost ecological planting methods (El Shaer, 2010). Relevant research shows that the rational development and use of saline and alkaline land can not only provide a stable source of fodder for animal husbandry (Liang et al., 2013; Abebe and Tu, 2024), but also improve the ecological environment of saline and alkaline land through vegetation restoration, and further realize the comprehensive use value of land resources (Wang et al., 2022).

*Potentilla anserina* Linnaeus (*P. anserina*), a member of the Rosaceae family, is a medicinal plant with a long history of use in Tibetan medicine in China (Li et al., 2024). Its swollen tuberous root is a medicinal food known as “jue ma” in Chinese (Wang et al., 2010). The *P. anserina* has strong characteristics of cold, drought and salinity, can grow well in barren land and alpine areas, ecologically fragile areas of grassland restoration and land use is of great importance (Cheng et al., 2022). Its nutrients and diverse metabolite composition have the potential to be exploited as a new feed resource (Guo et al., 2023). Rich in crude protein, starch and a variety of trace elements, *P. anserina* can provide a complete diet for livestock (Jiao et al., 2025). At the same time, however, although is rich in nutrients and has therapeutic effects on many diseases, its use as a forage in salt-alkaline soils and related research are relatively scarce. The development of fern forage for saline and alkaline environments can not only alleviate the problem of forage resource scarcity, but also provide an effective way to improve saline and alkaline soils, which is an area worthy of in-depth exploration and research.

Therefore, this study investigated dry, silage and fresh samples of *P.anserina* from saline and normal areas to determine the nutritional quality and feeding characteristics of saline areas. Volatile and non-volatile compounds at different processing stages were analyzed using headspace solid-phase microextraction combined with gas chromatography–mass spectrometry (HS-SPME-GC-MS) and high-performance liquid chromatography–mass spectrometry (HPLC-MS), respectively. Microbial community dynamics of *P. anserina* across various stages of processing were characterized through amplicon sequencing. The influence of salinity on processing-induced changes and microbial behavior was systematically examined. This research offers meaningful insights into how salinity affects the processing of *P. anserina*, and contributes valuable data for future studies related to the utilization of saline-alkali land and the functional development of this forage plant.

## 2 Materials and methods

### 2.1 Plant collection, air-dried and ensiling

**Figure 1:**
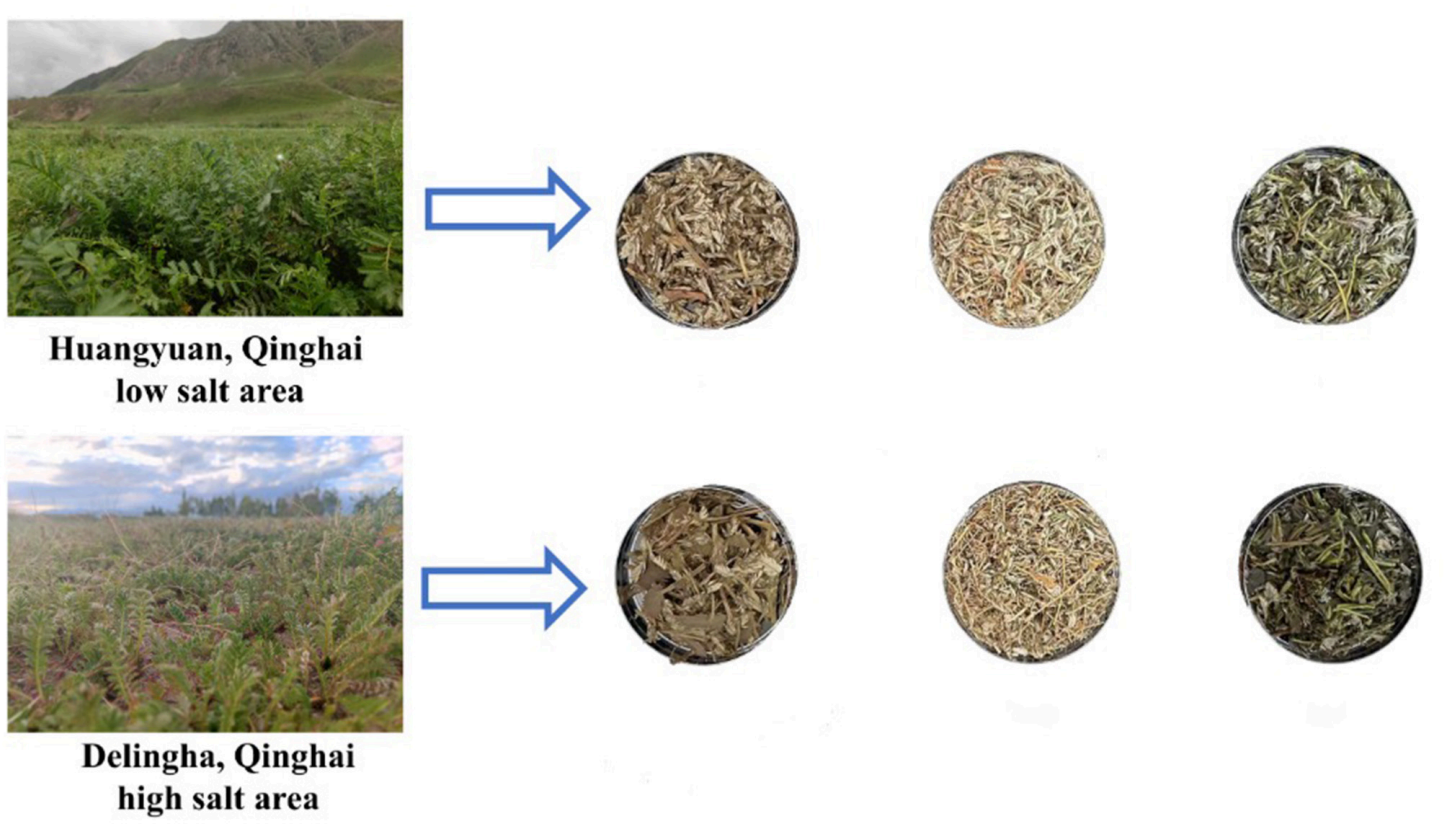
Processing of silage, dry and fresh samples in high and low salt areas figure. HF represents the low-salt fresh samples, HD represents the low-salt dry grass, HS represents the low-salt silage, DF represents the high-salt fresh samples, DD represents the high-salt dry grass, and DS represents the high-salt silage.

*Potentilla anserina* linnaeus (*P. anserina*) was collected on 28 July 2024 at two sites in Qinghai Province, China. Stem and leaves of *P. anserina* cultivar “Qinghai No. 6” were collected from a saline-alkali soil site in Delingha (D06; 37.2084°N, 97.3988°E) and a non-high-salt site in Huangyuan (H06; 36.5260°N, 101.1338°E). During the full bloom period of *P. anserina*, fresh leaves (HF) were harvested when the stubble height is 2 cm *P. anserina* was selected because of its use in Chinese medicine and its potential value as a functional feed supplement for ruminants. After collection of fresh material, sunlight was used to produce 30% dry matter before the leaves were chopped to a size of 2 cm (Li et al., 2022). Samples of 300 g, in triplicate, were placed in polyethylene plastic bags (dimensions 25 cm × 35 cm, China) and then vacuum sealed (45 d), producing 3 bags of normal silage samples (HS) and 3 bags of saline land silage samples (DS), while the other half was air-dried into hay, producing normal hay samples (HD) and saline land hay samples (DD), and stored together at room temperature (25–30 °C) (Figure 1).

### 2.2 Quality analysis

The chemical composition of hay and silage samples was analyzed after milling. Crude protein content was determined using the Dumas nitrogen analysis method (Model Dumatherm 7700, Gerhardt Analytical Instruments Co. Ltd., Germany). An ANKOM fiber analyser (Model A2000i, ANKOM Technology, Macedon, NY, USA) was used to quantify crude fiber (CF), acid detergent fiber (ADF) and neutral detergent fiber (NDF). Crude fat analysis was performed using an ANKOM fat analyser (Model XT15i, Beijing ANKOM Technology Co. Ltd., China). Ash content was measured using the VAOAC International Official Methods of Analysis method (Thiex et al., 2012).

### 2.3 Determination of volatile compounds

To investigate the volatile constituents in *P. anserina*, headspace solid-phase microextraction combined with gas chromatography-mass spectrometry (HS-SPME-GC-MS) was utilized. Precisely 2 grams of *P. anserina* powder were placed in a 20 mL headspace vial, followed by the addition of 10 μL of an ethyl decanoate internal standard solution (0.2155 g/L). For each *P. anserina* type, three replicate samples were prepared for analysis. Volatile compounds were extracted using a 50/30 μm DVB/CAR/PDMS SPME fiber (Sigma-Aldrich, St. Louis, MO, USA) at 60 °C for a duration of 30 min. The fiber was then inserted into the injection port of the GC-MS system and desorbed at 250 °C for 5 min. Chromatographic separation and detection were carried out using a Trace1310 GC and TSQ8000 MS (Thermo Scientific, Waltham, MA, USA), employing a TG-5 capillary column (60 m × 0.25 mm × 0.25 μm, Thermo Scientific). Helium served as the carrier gas at a constant flow rate of 1 mL/min, and the injector temperature was maintained at 270 °C. The oven program began at 40 °C (held for 2 min), ramped at 10 °C/min to 250 °C, and held for 5 min. The temperatures for the transfer line and ion source were set at 280 °C and 300 °C, respectively. Mass spectrometry operated under electron impact ionization at 70 eV, scanning a mass range from m/z 33 to 400. Volatile compounds were identified based on retention indices (RI), calculated using a standard series of n-alkanes (C7–C30), and verified against the NIST spectral database. Quantification was carried out semi-quantitatively using ethyl decanoate as the sole internal standard.

### 2.4 Analysis of non-volatile metabolites

A broadly targeted metabolomic analysis of *P. anserina* was carried out using an LC-MS/MS system from Shanghai Parsonage Biotechnology Co. The freeze-dried samples were crushed with beads for 30 s at 60 Hz. 25 mg aliquot of individual samples were accurately weighed and were transferred to an Eppendorf tube. 1000 μL of extract solution (methanol/water = 3:1, containing internal standard) and beads were added. After 30 s vortex, the mixed samples were homogenized (40 Hz, 4 min) and sonicated for 5 min in 4 °C water bath, the step was repeated for three times. Then the samples were extracted over night at 4 °C on a shaker. Then samples were centrifuged at 12,000 rpm for 15 min at 4 °C. The supernatant was carefully filtered through a 0.22 μm microporous membrane and transferred to 2 mL glass vials. 0.33 μL from each sample was taken and pooed as QC samples.

The UHPLC separation was carried out using an EXIONLC System (Sciex). The mobile phase A was 0.01% acetic acid in water, and the mobile phase B was 50% ACN/IPA. The column temperature was set at 25 °C. The autosampler temperature was set at 4 °C and the injection volume was 2 μL. The flow rate was 0.3 mL/min. A Sciex QTrap 6500+ (Sciex Technologies), was applied for assay development. Typical ion source parameters were: IonSpray Voltage: 5500 V/−4500 V, Curtain Gas: 35 psi, Temperature: 400 °C, Ion Source Gas 50 psi.

### 2.5 DNA extraction and amplification sequencing

Three 10 g samples of *P. anserina* collected from both low-salinity and high-salinity environments were ground into fine powder under liquid nitrogen conditions. The resulting powder was suspended in 100 ml of sterile saline solution and shaken at 10 °C and 220 rpm for 3 h. The mixture was then filtered through eight layers of sterile gauze, and the filtrate was centrifuged at 1307 × g for 15 min at 4 °C to collect the pellet. The obtained precipitates were stored at −80 °C. Each sample was processed in triplicate. Total DNA extraction was carried out using the OMEGA Soil DNA Kit (D5625-01, Omega Bio-Tek, Norcross, GA, USA). DNA concentration was measured with a Nanodrop spectrophotometer, and DNA integrity was checked via 1.2% agarose gel electrophoresis.

For sequencing, the microbial DNA from *P. anserina* samples was subjected to paired-end sequencing. The bacterial 16S rRNA V4–V5 region was amplified using universal primers 515F (5′-GTGCCAGCMGCCGCGGTAA-3′) and 907R (5′-CCGTCAATTCMTTTRAGTTT-3′), while the fungal ITS1 region was amplified using ITS5F (5′-GGAAGTAAAAGTCGTAACAAGG-3′) and ITS2R (5′-GCTGCGTTCTTCGATCGTC-3′). Each primer pair was labeled with a unique 7-bp barcode for multiplex sequencing. The resulting PCR products were purified using VAHTSTM DNA Clean Beads (Vazyme, Nanjing, China), and their concentrations were determined using the Quant-iT PicoGreen dsDNA Assay Kit (Invitrogen, Carlsbad, CA, USA). Equimolar amounts of amplicons were pooled and subjected to sequencing on an Illumina MiSeq platform with the MiSeq Reagent Kit v3, generating fragments ranging from 200 to 450 bp, conducted by Shanghai Personal Biotechnology Co., Ltd. (Shanghai, China).

Microbial community analysis was conducted using QIIME2 (version 2023.9) (Zhu et al., 2022). Raw sequences were first demultiplexed using the Demux plugin, followed by primer trimming with Cutadapt. DADA2 was applied for sequence quality control, including filtering, denoising, merging of reads, and chimera removal. High-quality sequences were then taxonomically assigned by alignment against the NCBI database for bacterial and fungal identification. Downstream analyses, including alpha diversity metrics, Bray–Curtis-based principal coordinate analysis (PCoA), and assessment of dominant microbial taxa, were performed in RStudio (version 4.2.2) using the “vegan”, “plyr”, and “ggplo2” packages.

### 2.6 Statistical analysis

All statistical analyses were carried out using SPSS software (version 19.0; SPSS Inc., Chicago, IL, USA). Identification of differential metabolites was based on two criteria: a variable importance in projection (VIP) score of ≥1 and an absolute Log2 fold change (|Log2FC| ≥ 1.0). VIP scores were derived from orthogonal partial least squares discriminant analysis (OPLS-DA), which also produced both score plots and permutation plots. These visualizations and analyses were conducted using the MetaboAnalystR package in R. Prior to OPLS-DA, the data underwent log2 transformation and were mean-centered. To assess the robustness of the OPLS-DA model and minimize the risk of overfitting, a permutation test with 200 permutations was performed.

## 3 Results

### 3.1 Chemical composition of in low-salt and high-salt habitats

In this study, the chemical compositions of fresh grass, hay and silage under low-salt and high-salt habitats were compared (Table 1). This study found that the crude protein (CP) content in saline-alkali soil (9.57% DF; 9.36% DD; 11.22% DS) was lower than in normal soil (12.80% HF; 12.80% HD; 14.27% HS) (*P* < 0.05), particularly in hay. However, the CP content increased after ensiling, regardless of whether the soil was normal or saline-alkali (*P* < 0.05). The levels of crude fiber(CF; 12.61% HF; 16.11% HD; 16.06% HS; 9.57% DF; 9.36% DD; 11.22% DS), neutral detergent fiber (NDF; 37.57% HF; 32.48% HD; 38.19% HS; 30.63% DF; 29.74% DD; 34.42% DS) and acid detergent fiber (ADF; 29.42% HF; 27.83% HD; 28.21% HS; 24.82% DF; 25.04% DD; 27.36% DS) in saline-alkali soil are all lower than in normal soil (*P* < 0.05). The content of ether extract (EE) is higher under saline-alkali (38.33% DF; 30.05% DD; 38.00% DS) conditions than under normal soil conditions (35.80% HF; 23.69% HD; 33.13% HS). Furthermore, the EE content in saline-alkali soil after silage is not significantly different from that in fresh leaves (*P* > 0.05). The ash content of the other treatments was found to be significantly different from that of HF (15.57%) (*P* < 0.01).

**Table 1:** Nutritional quality of *P. anserina* stems and leaves under different treatments.

**Treatments**	**CP(%DM)**	**CF(%DM)**	**Ash(%DM)**	**NDF(%DM)**	**ADF(%DM)**	**EE(%DM)**
HF	12.80c	12.61c	15.57a	37.57a	29.42a	35.80ab
HD	13.72b	16.11a	9.65d	32.48c	27.83bc	23.69d
HS	14.27a	16.06a	10.46b	38.19a	28.21b	33.13bc
DF	9.57e	11.68d	9.56d	30.63d	24.82d	38.33a
DD	9.36f	15.53b	9.51d	29.74d	25.04d	30.05c
DS	11.22d	15.71ab	10.10c	34.42b	27.36c	38.00a

Different letters indicate significant differences between treatments. HF, low-salt fresh sample; DF, high-salt fresh sample; HD, low-salt hay sample; DD, high-salt hay sample; HS, low-salt silage sample; DS, high-salt silage sample; CP, crude protein; CF, crude fiber; Ash, Crude ash; EE, ether extract; ADF, acid detergent fiber; NDF, neutral detergent fiber.

### 3.2 Full mass spectrometric analysis of volatile metabolites of in low-salt and high-salt habitats

**Figure 2:**
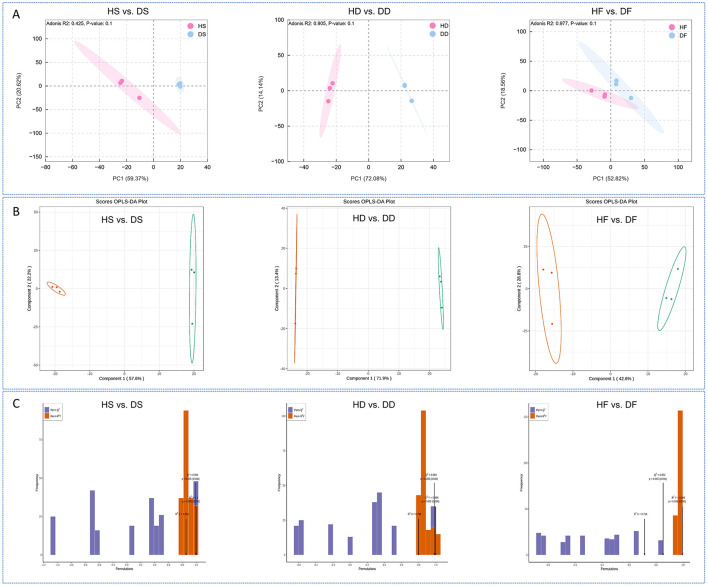
Princi *P. anserina* component analysis **(A)**, OPLS-DA score plots **(B)** of volatile metabolites for the comparison groups, validation plots of the OPLA-DA model for volatile metabolites in the *P. anserina* **(C)**. HF represents the low-salt fresh samples, HD represents the low-salt dry grass, HS represents the low-salt silage, DF represents the high-salt fresh samples, DD represents the high-salt dry grass, and DS represents the high-salt silage.

In this research, metabolomic profiling was conducted to assess the qualitative and quantitative differences between low-salt and high-salt *P. anserina* samples. A comprehensive analysis identified 996 volatile metabolites were detected, including 233 terpenoids, 168 ester, 124 ketone, 87 heterocyclic compound, 82 alcohol, 63 hydrocarbons, 59 aldehyde, 49 phenol, 45 acid, 24 aromatics, 23 amineand, 17 Ether, 17 ether, 7 Halogenated hydrocarbons, and 3 sulfur compounds metabolites (Supplementary Data S1). The PCAmodels demonstrated that high-salt caused metabolic variations in the three treatments (Figure 2A). The OPLS-DA models exhibited high stability and reliability upon validation (Figure 2B). To prevent model overfitting, a 200-permutation test was performed prior to the OPLS-DA analysis. The constructed models demonstrated excellent predictive capability across all treatment groups. The respective R^2^X, R^2^Y and Q^2^ values were as follows: Silage treatment: HS vs. DS (R^2^X = 0.853, R^2^Y = 1, Q^2^ = 0.994); Dry treatment: HD vs. DD (R^2^X = 0.798, R^2^Y = 0.999, Q^2^ = 0.983); Fresh treatment: HF vs. DF (R^2^X = 0.714, R^2^Y = 0.995, Q^2^ = 0.852). All Q^2^ values exceeded 0.85, indicating robust model stability (Figure 2C). These findings collectively demonstrate that high-salt conditions significantly alter the volatile metabolite profiles of *P. anserina* when processed in different ways.

#### 3.2.1 Differential metabolite screening and class analysis of in low-salt and high-salt habitats

**Figure 3:**
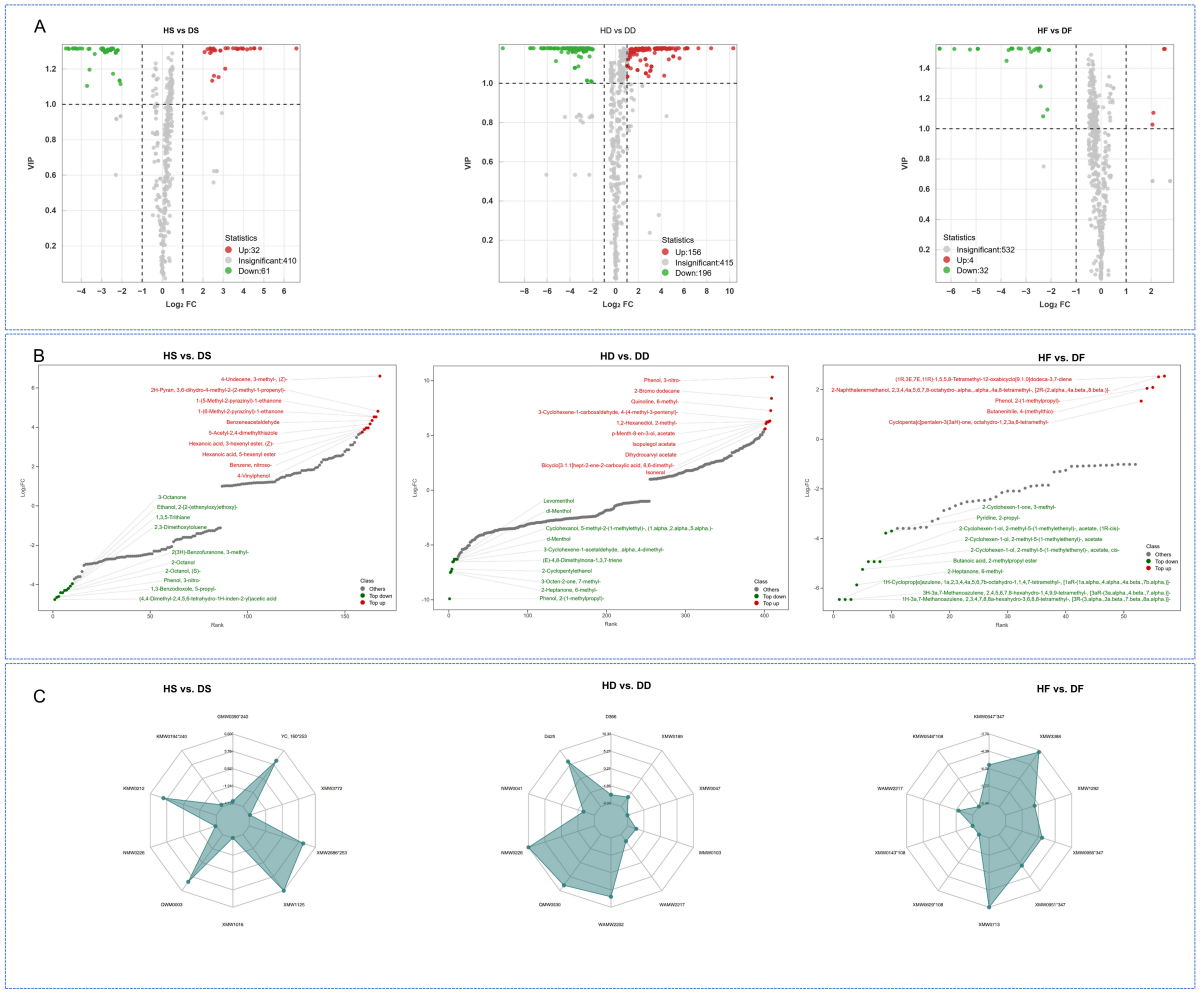
Volcano plot of the differential non-volatile metabolites of HD vs. DD, HS vs. DS, HF vs. DF **(A)**. Top FC distribution of nonvolatile metabolites **(B)**, radar chart of the top 10 (|log2FC|) nonvolatile metabolites **(C)**. In the C plots, the red and green dots represent the upregulated and downregulated nonvolatile metabolites, respectively. HF represents the low-salt fresh samples, HD represents the low-salt dry grass, HS represents the low-salt silage, DF represents the high-salt fresh samples, DD represents the high-salt dry grass, and DS represents the high-salt silage.

We identified significant metabolic alterations across treatment groups using selection criteria of fold change (≥2 or ≤ 0.5) combined with VIP scores (≥1). This revealed 352 differentially abundant metabolites (DAMs) in HD vs. DD comparisons, 93 DAMs in HS vs. DS and 36 DAMs in HF vs. DF. Two metabolites showed consistent differential abundance across groups (Supplementary Data S2). Directional analysis revealed distinct regulatory patterns: the HS vs. DS comparison showed 61 downregulated and 32 upregulated DAMs, while the HD vs. DD comparison showed 196 downregulated and 156 upregulated DAMs. In contrast, the HF vs. DF comparison showed only 32 downregulated and 4 upregulated DAMs (Figure 3A). These 2 overlapping DAMs contained Phenol and Acid class of 1 and 1 metabolites, respectively. high-salt reduced the accumulation of these 2 overlapping DAMs in silage and hay *P. anserina*, however, in the processing of fresh leaves, the increase of Phenol, 2-(1-methylpropyl)- was promoted, while the acetic acid (4, 4-dimethyl-2,4,5,6-tetrahydro-1H-inden-2-yl) was reduced (Supplementary Data S2). The metabolite content dynamic distribution plot and radar chart showed the metabolites of the Top Fc of HS vs. DS, HD vs. DD and HF vs. DF caused by saline and alkaline stress (Figures 3B, C). In high-salt, the most significant upregulated metabolites in fresh leaves were (1R,3E,7E,11R)-1,5,5, 8-tetramethyl-12-oxabicyClo [9.1.0]dodeca-3,7-diene, The most significant down-regulated metabolites were 1H-3a, 7-methanoazulene, 2,3,4,7,8, 8-a-hexahydro-3,6,8,8-tetramethyl- [3 r—(3) alpha), 3 a. Beta., 7. Beta., 8 a. alpha.)]. After silage, we found that the most significantly down-regulated metabolites were (4, 4-dimethyl-2,4,5,6-tetrahydro-1H-inden-2-yl)acetic acid, 1, 3-benzodioxole, 5-propyl- and PhenoL. The most significantly up-regulated metabolite of 3-nitro- was 4-Undecene, 3-methyl-, (Z)-. After natural air-drying, we found that the most significantly down-regulated metabolite was Phenol, 2-(1-methylpropyl)-, 2-Heptanone, 6-methyl-. The most up-regulated metabolites are Phenol, 3-nitro-, Isopulegol acetate and Dihydrocarvyl acetate. We found that in HD and DD, the down-regulation of Phenol, 2-(1-methylpropyl)- was the most obvious, while in HS and DS, its up-regulation was significant. We found that Phenol, 3-nitro- decreased significantly during the silage process, while it was the metabolite that increased most significantly during the natural air-drying process. This might be worth our further study of the differences between silage and hay feed.

The compounds with ROAV ≥ 1 were screened; these 18 aldehydes, 16 ketone, 13 terpenoids, 8 phenol, 7 ester, 6 heterocyclic compound, 5 alcohols, 7 ester, 2 nitrogen compounds, 1 halogenated hydrocarbons and 1 sulfur compounds (Data S3) can be considered as the main contributors to the aroma of *P. anserina*. 1-Nonen-3-one (pungent, mushroom), 2 (5H)—Furanone,5-ethyl-3-hydroxy-4-methyl -(sweet, fruity, caramel, maple, fenugreek, brown, sugar, nutty, chicory, praline, butterscotch), 3-octen-2-one (earthy, spicy, herbal, sweet, mushroom, hay, blueberry), 1-octen-3-one (mushroom), and Dodecane nitrile (fruity, floral, berry, plum, black currant, honey, rose, tobacco) showed higher rOAV in all 5 treatments, indicating that these volatiles may contribute to a stronger aroma of *P. anserina*. Most of them are ketone compounds. According to our findings, 1-Nonen-3-one is regarded as the substance that contributes the most to the flavor characteristics of *P. anserina* in high-salt. Due to the high concentration and ROAV in DS samples (315,239.31 μg/g and 15,448.24, respectively), it may be regarded as an indicator of lipid oxidation in *P. anserina*.

#### 3.2.2 Full mass spectrometric analysis of nonvolatile metabolites in under low-salt and high-salt habitats

**Figure 4:**
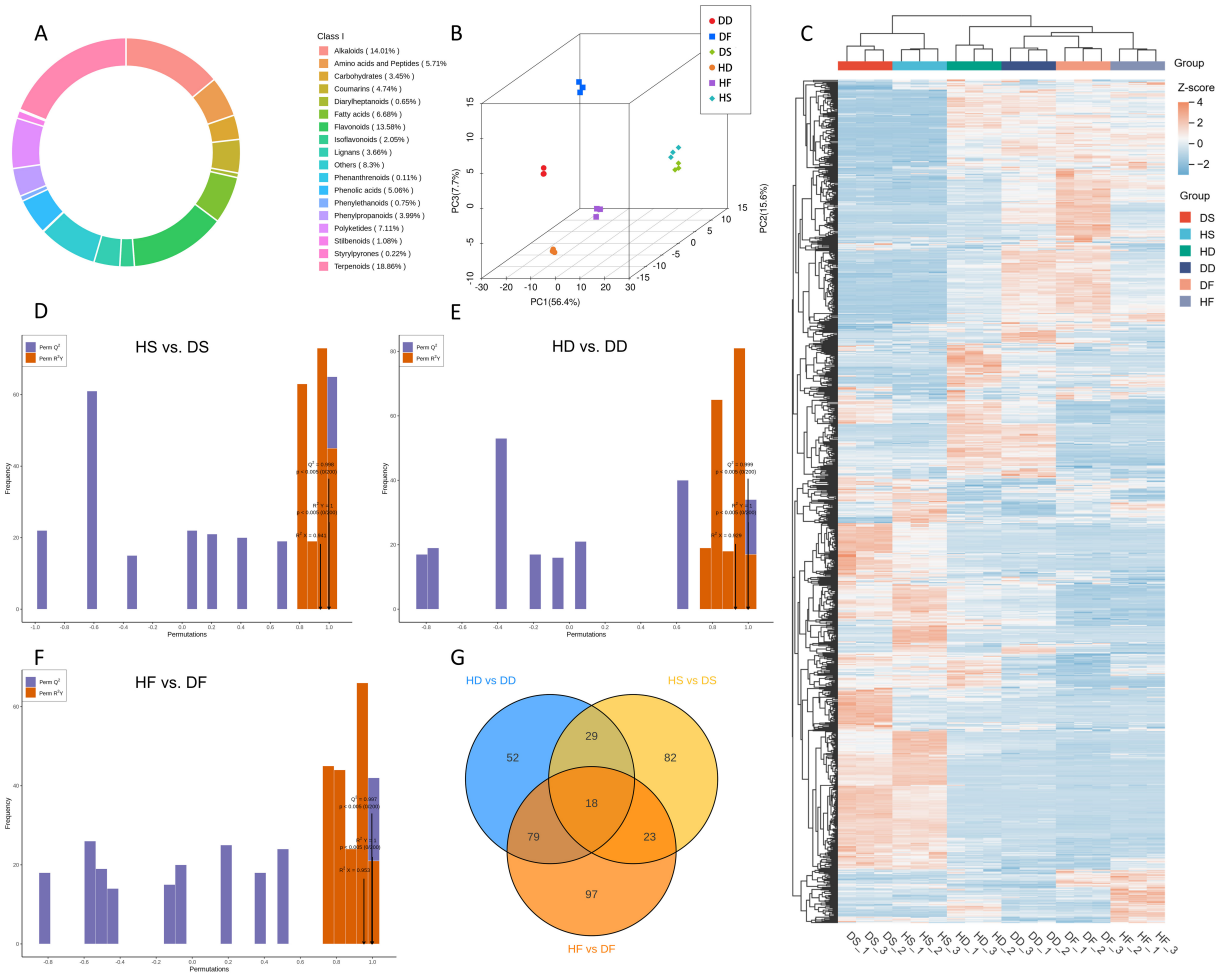
Ring chart of the proportion of all non-volatile metabolite categories **(A)**, Princi *P. anserina* component analysis **(B)**, Heat map of metabolite levels in *P. anserina* from different processes **(C)**, validation plots of the OPLA-DA model for non-volatile metabolites in the HS vs. DS **(D)**, validation plots of the OPLA-DA model for non-volatile metabolites in the HD vs. DD **(E)**, validation plots of the OPLA-DA model for non-volatile metabolites in the HF vs. DF **(F)**, Venn diagram of differential nonvolatile metabolites for the comparison groups **(G)**. HF represents the low-salt fresh samples, HD represents the low-salt dry grass, HS represents the low-salt silage, DF represents the high-salt fresh samples, DD represents the high-salt dry grass, and DS represents the high-salt silage.

In this study, 928 non-volatile metabolites were detected (Figure 4A), including 175 terpenoids, 126 flavonoids, 130 alkaloids, 78 lignans and coumarins, 66 polyketides, 62 fatty acids, 53 amino acids and derivatives, 47 phenolic acids, 37 phenylpropanoids, 32 carbohydrates, 19 isoflavonoids, 10 stilbenoids, 7 phenylethanoids, 6 diarylheptanoids, 2 styrylpyrones, 1 phenanthrenoids, and 77 other metabolites (Data S4). The contribution rates of the three princi *P. anserina* components (PC1, PC2, and PC3) obtained from the princi *P. anserina* component analysis (PCA) score plot (including QC) were 56.4, 15.6, and 7.7%, respectively (Figure 4B). We found significant differences between *P. anserina* fresh samples, hay and silage. However, after silage, the difference between saline-alkali stress and normal condition became smaller, while there was still significant difference between fresh grass and hay. This was also confirmed by the cluster heat map of the metabolites of the sample (Figure 4C).

To prevent model overfitting, a 200-permutation test was performed prior to the OPLS-DA analysis. The constructed models demonstrated excellent predictive capability across all treatment groups. The respective R2X, R2Y and Q2 values were as follows: Silage treatment: HS vs. DS (R2X = 0.941, R2Y = 1, Q2 = 0.998); Dry treatment: HD vs. DD (R2X = 0.929, R2Y = 1, Q2 = 0.999); Fresh treatment: HF vs. DF (R2X = 0.953, R2Y = 1, Q2 = 0.997). All Q2 values exceeded 0.85, indicating robust model stability (Figures 4D–F). These findings collectively demonstrate that high-salt conditions significantly alter the volatile metabolite profiles of *P. anserina* when processed in different ways.

#### 3.2.3 Differential compound screening of in low-salt and high-salt habitats

**Figure 5:**
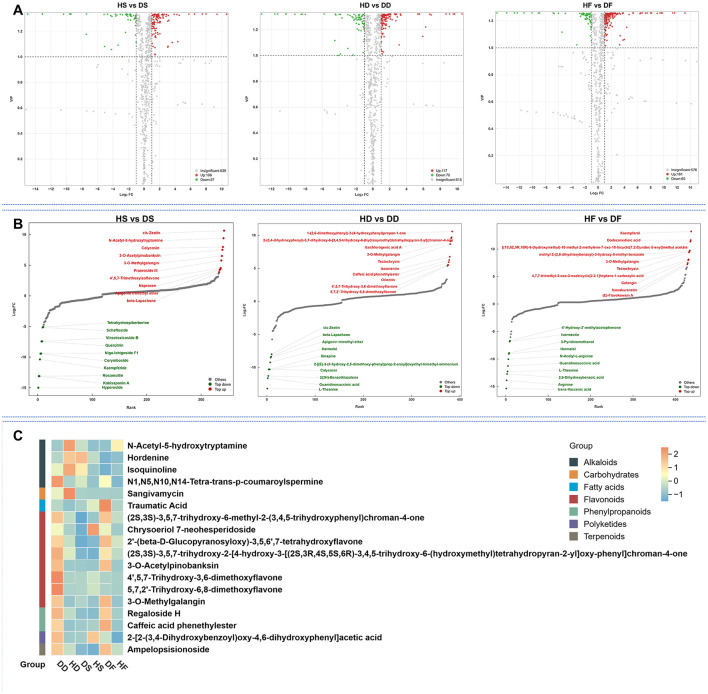
Volcano plot of the differential non-volatile metabolites of HD vs. DD, HS vs. DS, HF vs. DF **(A)**, TopFC distribution of the differential non-volatile metabolites of HD vs. DD, HS vs. DS, and HF vs. DF **(B)**, Heat map of different levels of volatile metabolites under normal conditions and alkaline stress **(C)**. HF represents the low-salt fresh samples, HD represents the low-salt dry grass, HS represents the low-salt silage, DF represents the high-salt fresh samples, DD represents the high-salt dry grass, and DS represents the high-salt silage.

A total of 344 differential metabolites (DAMs) were identified between the HS and DS groups using criteria of fold change ≥ 2 or ≤ 0.5, along with VIP scores ≥ 1. By comparison, 384 DAMs were found in the HD vs. DD analysis. The HF vs. DF analysis detected 437 DAMs, 18 of which were shared across the comparisons (Figure 4G). High-salt was shown to affect 18 different metabolites in common in the three comparison groups. These 18 overlapping DAMs contained 8 flavonoids, 4 alkaloids, 2 phenylpropanoids, 1 carbohydrates, 1 polyketides 1 fatty acids, and 1 terpenoids class metabolites, respectively. Among these overlapping DAMs, for hay and fresh, both salt stress and alkaline stress promoted the accumulation of 14 and 15 DAMs in the *P. anserina*, and only the accumulation of N-Acetyl-5-hydroxytryptamine, hordenine, and isoquinoline were reduced by saline and alkaline stress. However, for silage, high-salt enhanced the accumulation of 10 DAMs, reduced the accumulation of 8 DAMs, the most up-regulated differential metabolite was N-Acetyl-5-hydroxytryptamine and the most down-regulated differential metabolite was Chrysoeriol 7-neohesperidosid (Supplementary Data S2). A total of 57 downregulated and 106 upregulated differential metabolites (DAMs) were observed in the HD vs. DD group. In the HD vs. DD comparison, 70 DAMs were found to be downregulated and 117 to be upregulated. In the HF vs. DF group, 65 DAMs were found to be downregulated and 161 to be upregulated (Figure 5A). The distribution charts of metabolite dynamics revealed the metabolites that were most significantly altered (top Fc) in hay, silage and fresh samples that were subjected to saline-alkaline stress (Figure 5B). Of the metabolites found in hay, 3-O-methylgalangin was the most significantly upregulated compound, while 2-[(E)-3-(4-hydroxy-2,5-dimethoxyphenyl)prop-2-enoyl]oxyethyl trimethylammonium was the most notably downregulated. Silage samples showed the highest degree of upregulation for cis-zeatin, whereas hyperoside exhibited the most significant downregulation. Kaempferol acid was the most significantly up-regulated metabolite in fresh, while trans-Vaccenic acid was the most significantly down-regulated. To more intuitively observe the difference in metabolites in stems and leaves of *P. anserina* under high-salt, we drew heat maps of non-volatile metabolites under different treatments (Figure 5C). The effects of high-salt in the *P. anserina* of the three treatments were not completely consistent.

### 3.3 Microbiological analysis of in low-salt and high-salt habitats

**Figure 6:**
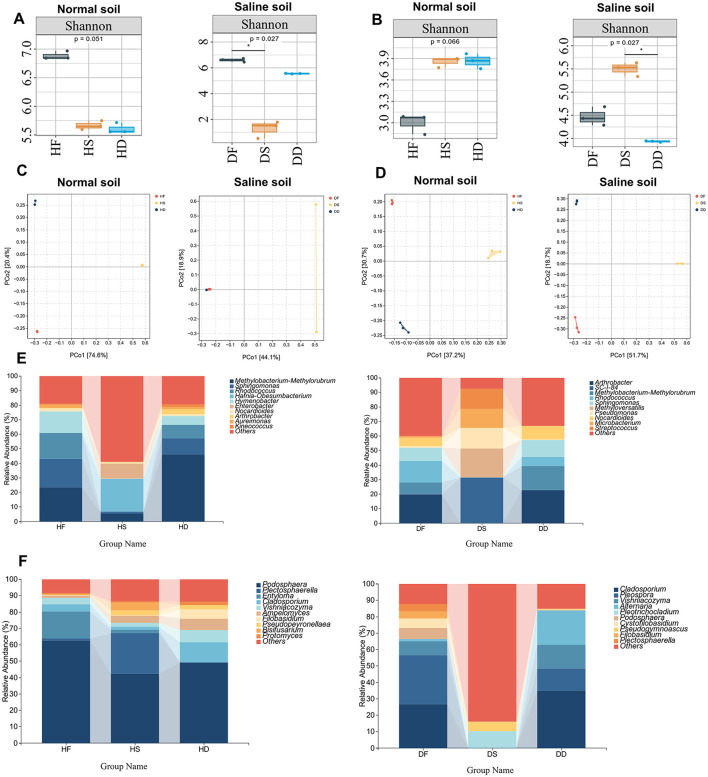
Microbial analysis of fresh leaves, hay, and silage stages of *P. anserina* in different production areas. Bacterial α-diversity analysis (Shannon Index) **(A)**; Fungal α-diversity analysis (Shannon Index) **(B)**; Bacterial PCoA analysis based on Bray-Curtis distance **(C)**; Fungal PCoA analysis based on Bray-Curtis distance **(D)**; Dominant bacterial genus analysis **(E)**; Dominant fungal genus analysis **(F)**. HF represents the low-salt fresh samples, HD represents the low-salt dry grass, HS represents the low-salt silage, DF represents the high-salt fresh samples, DD represents the high-salt dry grass, and DS represents the high-salt silage.

In this study, microorganisms were detected by 16S rRNA and ITS amplicon sequencing from fresh, hay, and silage samples to analyse the effect of microbial activity on the stem and leaf parts of, and to analyse changes and differences in microbial community diversity and structure of stem and leaf parts by salinity on different treatments at the genus level. To analyse the α-diversity of microbial communities in different samples, the Shannon index was used to reflect the richness and evenness of microorganisms. As shown in Figure 6A, analysis of the microbiome diversity index of different samples showed that processing reduced biodiversity (*P* < 0.05). In particular, in saline alkaline soil, the α-diversity of DS is the lowest. As can be seen from Figure 6B, in low-salt, the abundance of stems and leaves after treatment was higher than that of HF fungi, while in saline-alkali soil, DD was higher than that after DF treatment (*P* < 0.05). Overall, the α-diversity of bacteria was significantly higher in low-salt compared to high-salt, while the α-diversity of bacteria was relatively lower in high-salt compared to low-salt. These findings suggest that bacterial abundance and evenness decreased under high-salt conditions, while fungal abundance and evenness increased. Microbial activity in high-salt environments may be associated with *P. anserina*. Across both low- and high-salt conditions, the lowest bacterial α-diversity was observed in the DS group, while the remaining five samples exhibited higher bacterial α-diversity. In contrast, the highest fungal α-diversity was observed in the DS group, suggesting that fungi may exert a greater influence on quality than bacteria in this environment. Conversely, bacterial communities may play a more significant role in determining quality in HF, HD, HS, DF and DD. To explore β-diversity, a Principal Coordinates Analysis (PCoA) based on Bray–Curtis distances was performed (Figures 6C, D), revealing clear clustering of both bacterial and fungal communities under low- and high-salt conditions. We found that, whether in low-salt or high-salt, except for the bacteria DD and DF under high-salt, there were significant differences in bacteria and fungi under different treatments (*P* < 0.05). In summary, the bacterial and fungal genera present in in normal and high-salts show certain geographical differences, which become more pronounced after processing.

In order to gain a deeper insight into the differences in the structure of bacterial communities associated with *P. anserina* in non-saline versus saline conditions across fresh, hay and silage samples, we examined shifts in the composition and relative abundance of the 10 most dominant bacterial genera. As shown in Figure 6E, The dominant genus in the HF group was *Methylobacterium-Methylorubrum* (23.44%), followed by *Sphingomonas* (19.78%), *Rhodococcus* (17.80%) and *Hymenobacter* (14.66%). The dominant genus in the DF group was *Arthrobacter* (19.86%), followed by *Rhodococcus* (14.83%), *Sphingomonas* (9.03%) and *Methylobacterium-Methylorubrum* (8.20%). The dominant genus in the HS group was *Hafnia-Obesumbacterium* (22.21%), followed by *Methylobacterium-Methylorubrum* (5.61%). The dominant genus in the DS group was *SC-I-84* (31.43%), followed by *Methyloversatilis* (19.95%) and *Pseudomonas* (14.11%). The dominant genus in the HD group was *Methylobacterium-Methylorubrum* (46.09%), followed by *Sphingomonas* (11.08%) and *Rhodococcus* (9.32%). The dominant genus in the DD group was *Arthrobacter* (22.68%), followed by *Methylobacterium-Methylorubrum* (16.75%), *Sphingomonas* (11.48%) and *Rhodococcus* (6.21%). The capabilities of *Methylobacterium-Methylorubrum* include the synthesis of auxin, gibberellin, cytokinin and 1-aminocyclopropane-1-carboxylate (ACC) deaminase, as well as nitrogen fixation (Zhang et al., 2024).

The relative abundance composition of the dominant fungal genera is shown in Figure 6F. The dominant genus in the HF group was *Podosphaera* (62.46%), followed by *Entyloma* (16.56%), *Cladosporium* (4.21%) and *Vishniacozyma* (23.44%). the dominant genus in the DF group was *Pleospora* (29.87%) followed by *Cladosporium* (26.61%), *Vishniacozyma* (8.53%) and *Podosphaera* (6.65%). The dominant genus in HS group was *Podosphaera* (42.51%) followed by *Plectosphaerella* (24.68%), *Bisifusarium* (4.78%) and *Ampelomyces* (4.40%).The dominant genus in DS group was *Pleotrichocladium* (10.34%).The dominant genus in HD group was *Podosphaera* (49.07%) followed by *Cladosporium* (12.06%), *Vishniacozyma* (7.61%) and *Ampelomyces* (6.74%).The dominant genus in the DD group was *Cladosporium* (35.06%) followed by *Alternaria* (20.79%), *Vishniacozyma* (14.53%) and *Pleospora* (13.43%). We found that *Podosphaera* was the dominant genus in all treatments under low-salt conditions and HS and HD were significantly less than HF (*P* < 0.05). However, *Podosphaera* spp. were significantly lower (*P* < 0.05) under high-salt conditions and largely disappeared under DS and DD treatments. Whereas, *Cladosporium* bacilli increased significantly (*P* < 0.05) under high-salt conditions. *Pleospora* bacillus appeared under high-salt conditions.

### 3.4 Correlation networks reveal microbial associations with volatile and non-volatile metabolites

**Figure 7:**
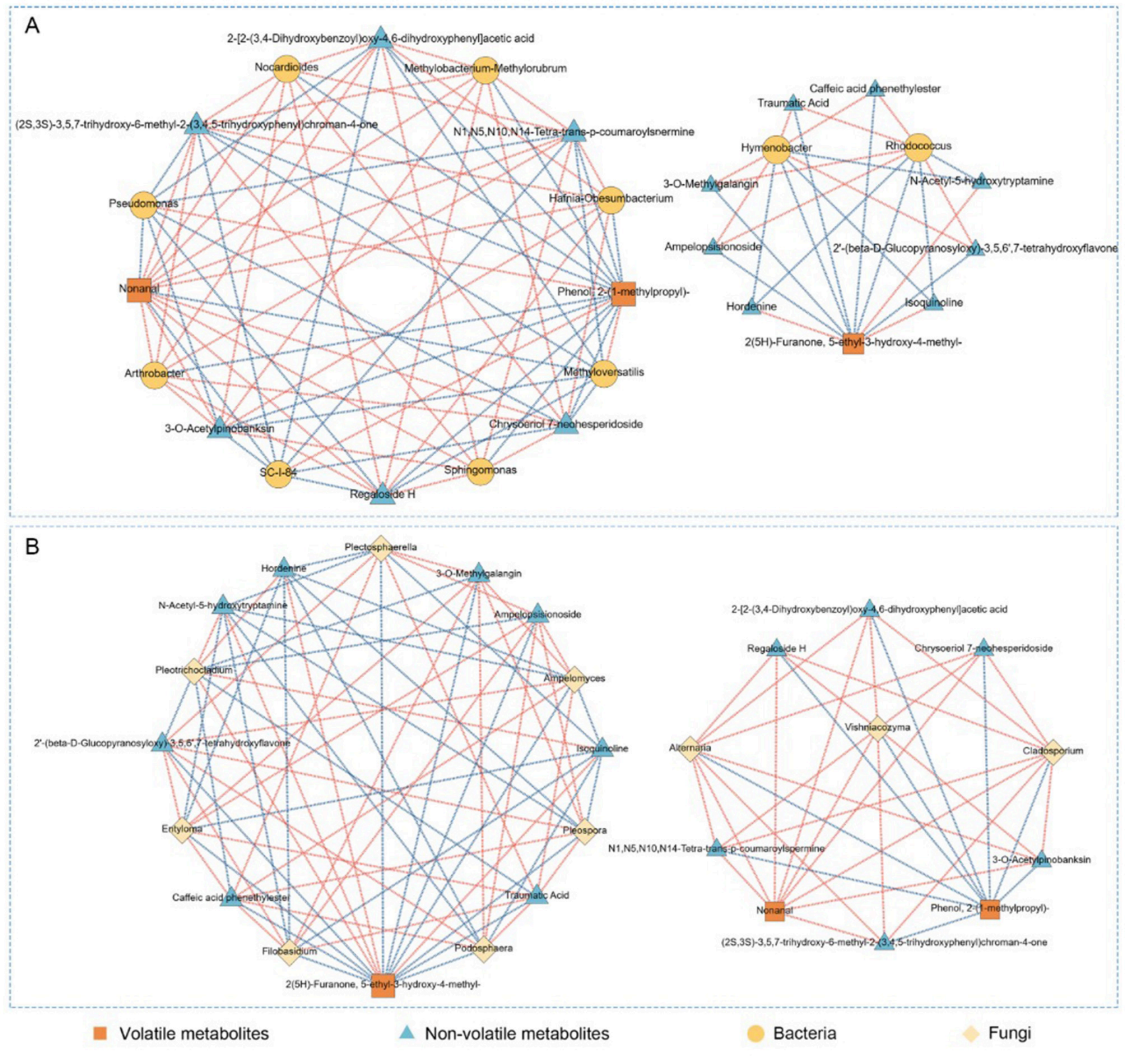
Correlation network analysis of bacteria **(A)** and fungi **(B)** in *P. anserina* with respect to volatile and non-volatile compounds. Where the red line represents significant positive correlation (ρ < 0.05, *r* > 0.6), the blue line represents significant negative correlation (ρ < 0.05, *r* < − 0.6), the thickness of the line represents the strength of the correlation; the size of the node represents the magnitude of the degree of connectivity. (For interpretation of the references to color in this figure legend, the reader is referred to the web version of this article).

To explore the associations between microbial communities and metabolite accumulation in *P. anserina* under high-salt treatments, we constructed Spearman correlation networks using metabolomics and microbiome data from DF, DD, and DS samples. The results are presented in Figure 7A (bacteria) and Figure 7B (fungi), showing distinct network structures and specificity in microbial–metabolite associations. As shown in Figure 7A, bacteria were strongly correlated with a range of both volatile and non-volatile metabolites. Genera such as *Arthrobacter, Pseudomonas*, and *Methyloversatilis* exhibited strong positive correlations with key volatile compounds, notably Nonanal and Phenol, 2-(1-methylpropyl)-, which are prominent in DS samples. In addition, furanone-type volatiles like 2(5H)-Furanone, 5-ethyl-3-hydroxy-4-methyl- also showed negative correlations with genera such as *Rhodococcus* and *Hymenobacter*. Non-volatile metabolites such as Regaloside H, Caffeic acid, and Isoquercitrin were correlated with both environmental and stress-tolerant bacterial taxa, including *Sphingomonas* and Methylobacterium. These compounds are often linked to antioxidant defense and plant stress responses, indicating that bacterial metabolism may influence the bioactive compound profile of *P. anserina* under saline storage conditions. In Figure 7B, fungal correlation networks show strong associations with both non-volatile and select volatile metabolites. Genera such as *Alternaria, Cladosporium*, and Podospora were significantly correlated with compounds like Phenol, 2-(1-methylpropyl)- and NonanaL. Meanwhile, flavonoid-related non-volatile metabolites such as Chrysoeriol-7-O-hesperidoside and Isoquercitrin were primarily associated with *Plectosphaerella* and *Filobasidium*. These findings suggest that fungi may exert influence through both enzymatic activity and plant–microbe interactions, contributing to the metabolic diversification observed in DS samples.

## 4 Discussion

### 4.1 Influences of low-salt and high-salt habitats on the chemical composition of *P. anserina*

Previous studies have been carried out on the salt-alkali tolerance of multiple forage crops, such as Medicago sativa, Lotus glaber, and kikuyugrass (Robinson et al., 2004); however, the results are inconsistent (Abebe and Tu, 2024). This study found that the content of crude protein (CP) was generally low in high-salinity habitats, especially in hay (9.36% DD), but increased after ensiling compared with fresh grass and hay. This may be related to improved protein utilization resulting from plant enzyme activity. Furthermore, EE content was significantly higher in fresh and silage forms (DF: 38.33%, DS: 38.00%), suggesting an increase in lipid accumulation under salt stress. Exposure of forage to high temperatures or dry air during hay making can result in severe lipid reduction (Serrapica et al., 2020). The increase in EE content in saline alkali land may be due to the accumulation of reactive oxygen species (ROS) in the body of *P. Anserina* (Hasanuzzaman et al., 2021). The ash content was also relatively low, suggesting that mineral uptake was limited under saline-alkali conditions. This is in line with the previous research findings (Prasad, 2022). Nevertheless, it is worth our discovery that the difference in ash content of the treated feed is not very significant. Furthermore, it was discovered that the CF, NDF, and ADF in saline-alkali soil were lower. This could be attributed to the fact that the slower growth rate influenced the leaf-to-stem ratio, thereby enhancing the quality of forage. A high leaf-to-stem ratio typically indicates a higher nutritional value of forage (Collins et al., 2017). Among the processing methods, silage (S) showed better nutrient retention in high saline soils. For example, DS had higher CP than DD and EE comparable to DF, suggesting that ensiling *P. anserina* may help to buffer the negative effects of salinity on forage quality. In contrast, the silage treatment (HS) had the highest CP (14.27%) and a relatively balanced fiber and mineral content on low salinity soils, reflecting optimal forage quality.

### 4.2 Influences of low-salt and high-salt habitats on volatile and non-volatile metabolite profiles in *P. anserina*

Previous studies have shown that abiotic stress can increase the production of volatile metabolites in tea plants (Zeng et al., 2019). The impact of alkaline stress on the volatiles of broomcorn millet grains exhibits variation among different varieties (Ma et al., 2023). Nevertheless, a deficiency of nutrients has been demonstrated to result in a reduction of the aroma components of tea leaves (Zhou et al., 2022). In the present study, high-salt restrained volatile metabolite accumulation in DD and DF treatment, but improved the accumulation of most volatile metabolites in DS treatment. Among the identified volatile metabolites, Phenol, 2-(1-methylpropyl)- plays a key role in the salt-stress response of *P. anserina*. Its dual function—contributing both to aroma and stress resistance—makes it a potential biomarker for quality control and functional enhancement of saline forage crops (Hasanuzzaman et al., 2022; Sharma et al., 2019). It was discovered that 1-Nonen-3-one is the predominant flavor substance in *P. anserina*, featuring mainly pungent and mushroom odors. It has also been identified in pea protein (Liu et al., 2023). Owing to its distinctive aroma properties, this compound has been extensively applied as a flavoring and fragrance agent in domains such as food, cosmetics, medicine, and perfume (Ortner et al., 2005). In conclusion, the effect of high-salt treatment on the volatile metabolites of *P. anserina* varies depending on the treatment method. These results suggest that *P. anserina* forage are capable of modulating the metabolome, which could potentially contribute to the development of new plant-based diets or feed additives for sustainable livestock production.

Most flavonoids, phenylpropanoids, polyketides, and terpenoids increased significantly in DD and DF when exposed to high-salt. Among them, flavonoids metabolites are the most abundant including 12 nonvolatile metabolites, such as 3-O-Methylgalangin, (2S,3S)-3,5,7-trihydroxy-2-[4-hydroxy-3-[(2S,3R,4S,5S,6R)-3,4,5-trihydroxy-6-(hydroxymethyl)tetrahydropyran-2-yl]oxy-phenyl]chroman-4-one, and 3-O-Acetylpinobanksin. Intriguingly, there was not much difference in the high-salt and non-high-salt silage treatments. The non-volatile metabolites that are jointly influenced by saline soil in the three groups are flavonoids metabolites. This may be due to how the microbial biodegradation and breakage of plant cells during ensiling could explain the decreasing concentration of some metabolites. Consequently, the hypothesis is that the accumulation of flavonoids may confer a degree of resistance to alkaline stress. It has been demonstrated that analogous outcomes have been attained for other stresses and species. The presence of naringin, prunin and kaempferol derivatives was detected in the leaves of sorghum seedlings when subjected to conditions of saline and alkali stress (Ma et al., 2023). Exposure to cadmium stress resulted in a 21.53% increase in total flavonoid content in basil plants, which may be attributable to the activation of their antioxidant system (do Prado et al., 2022). 3-O-Methylgalangin was the common significantly up-regulated differential metabolite in all three groups. 3-O-Methylgalangin was the common significantly up-regulated differential metabolite in all three groups and certainly contributed to the considerable increase in concentration in saline *P. anserina* forage. 3-O-Methylgalangin, a naturally occurring flavonoid derived from “Alpinia officinarum” and propolis, exhibits significant bioactivities, including antioxidant, anti-inflammatory, and anticancer properties (Hasanuzzaman et al., 2021). Studies have demonstrated its ability to scavenge free radicals, inhibit inflammatory mediators, and induce apoptosis in cancer cells (Campos et al., 2012; Parra et al., 2016). These findings suggest its potential as a therapeutic agent for oxidative stress-related diseases, chronic inflammation, and cancer, warranting further investigation for clinical applications (Echeverría et al., 2017).

Flavonoid metabolites, nature's potent non-enzyme antioxidants, play a crucial role in enhancing plants' ability to withstand environmental stress without the need for external enzymes. These secondary metabolites are instrumental in enabling plants to harness their inherent antioxidant capabilities, effectively safeguarding them against the adverse effects of abiotic stress (Ma et al., 2020). In summary, these results suggest that saline *P. anserina* can modulate the metabolome, which could contribute to the development of new plants as feed for sustainable livestock production.

### 4.3 Influences of low-salt and high-salt habitats on bacterial and fungal communities associated with *P. anserina*

We found that *Methylobacterium-Methylorubrum* was the dominant genus in all *P. anserina* samples except the DS group. As it is an aerobic bacterium, its content will decrease after silage fermentation. However, it increased during natural air drying. Typically, microorganisms belonging to this genus are neutrophilic, with their population dwindling as pH levels drop. Likewise, *Methylobacterium-Methylorubrum* has also been detected in high abundance in alfalfa silage and Sorghum-Sudangrass silage following 60 days of the ensiling process (Zhihao et al., 2022; Ogunade et al., 2018). However, to date its role in silage fermentation is less known. Under high-salt, the amount of *Methylobacterium-Methylorubrum* was lower than under normal conditions, which may be due to competition with *Arthrobacter* under salt and alkali stress. *Arthrobacter* is typically characterized by its nutritional diversity and ability to degrade natural and synthetic organic compounds (Zhihao et al., 2022). It also exhibits strong tolerance to drought and salt-alkali conditions (Batt, 2014). Furthermore, *Arthrobacter* is regarded as an indicator of hygiene quality in the dairy industry due to its presence in the microbiota of raw cow's milk (Sutthiwong et al., 2023), We found that Arthrobacter became the dominant genus in saline-alkali soil and existed in large quantities in soil tolerant of salinity and alkalinity. However, after ensiling, we discovered that the Arthrobacter genus disappeared and a new dominant genus, SC-I-84, emerged. This result merits further exploration. In the DS group, we found that *Methyloversatilis* bacteria unique to *SC-I-84* and *Methyloversatilis* bacteria. *Methyloversatilis*, as an important strain represented by Betaproteobacteria, shows various important roles in anaerobic degradation (Aranda et al., 2024). These results indicate that high-salts have different effects on fern stems and leaves under different treatments, and the emergence of new strains of bacteria after silage fermentation is worth further investigation.

According to previous relevant studies, powdery mildew (PM), caused by the obligate fungal pathogen *Podosphaera*, is the most reported destructive disease of cultivated cucurbits worldwide (Margaritopoulou et al., 2022). We found that *Podosphaera* spp. were abundant under normal conditions, whereas they were virtually absent under high-salt conditions, and that the relative abundance of *Podosphaera* spp. was significantly reduced after silage and natural air-drying treatments. *Cladosporium* was abundantly elevated in fresh leaves and hay under high-salt conditions. *Cladosporium* species are commonly isolated from soil and air samples (indoor and outdoor environments), however, some species have also been reported to be endophytic and to cause disease in humans, animals and plants (Schubert et al., 2007; Pereira et al., 2022). Whereas we found a highly significant reduction of *Cladosporium* bacteria in DS under high-salt conditions compared to other treatments, and a relatively high abundance of *Pleotrichocladium* bacteria was found in DS, which has recently been associated with enhanced drought resistance, salinity tolerance, and may be useful in the treatment of serious health problems such as neurodegenerative diseases and antibiotic resistance (Rodríguez Martín-Aragón et al., 2023; Xu et al., 2024). However, there are a large number of other fungi in its DS, which warrants further research on fern silage under high-salt conditions.

### 4.4 The effect of the correlation between the microbial community and volatile and non-volatile metabolites in salt silage of *P. anserina*

Based on Spearman correlation networks, we observed that specific bacterial genera, such as *Arthrobacter, Pseudomonas*, and *Sphingomonas*, were positively associated with key volatile compounds, particularly aldehydes and phenols like Nonanal and Phenol, 2-(1-methylpropyl)-. These associations suggest that bacterial metabolism plays a central role in shaping aroma-active profiles in high-salt silage, possibly via enzymatic degradation of lipids and phenylpropanoid-derived precursors (Zhang et al., 2023; Yang et al., 2025). In contrast, fungal genera including *Alternaria, Plectosphaerella*, and *Podospora* were primarily linked with non-volatile metabolites such as flavonoids and phenolic acids (Caffeic acid, Isoquercitrin, Chrysoeriol-7-O-hesperidoside). These fungi may influence host secondary metabolism either through direct enzymatic activity or via biotic stress induction (Pusztahelyi et al., 2015; Wu et al., 2021). The divergence in microbial–metabolite interaction patterns reflects a division of functional roles: bacteria contribute to aroma formation and fermentation processes, whereas fungi are more associated with antioxidant activity, defense-related metabolites, and metabolic stabilization. Interestingly, furanone-type volatiles, particularly 2(5H)-Furanone, 5-ethyl-3-hydroxy-4-methyl-, were linked to both bacterial and fungal taxa, indicating a potential shared ecological niche or metabolic co-dependence in furanone production (Xian et al., 2024). Such interactions reveal the complexity of microbial–metabolite networks and highlight the ecological coordination between microbial communities in response to high-salt stress during preservation. Overall, these results underscore the integrated role of microbial populations in modulating both the chemical composition and quality-related traits of *P. anserina* silage, providing insights into potential microbial targets for improving silage quality under saline conditions.

## 5 Conclusions

*P. anserina*, a salt-tolerant and nutrient-rich plant, holds significant potential as a novel forage crop in saline-alkaline environments. Multi-omics analysis showed that silage processing under high-salt conditions improved the retention of volatile aroma compounds (e.g., 1-Nonen-3-one, 2(5H)-Furanone) and non-volatile flavonoids (e.g., 3-O-Methylgalangin). Meanwhile, shifts in microbial communities—such as the enrichment of beneficial bacteria (e.g., Pseudomonas, SC-I-84) and suppression of pathogens like Podosphaera—enhanced the forage's biochemical quality and functional safety. Correlation network analysis revealed distinct microbial roles: bacteria contribute to aroma and fermentation, while fungi are associated with antioxidant compounds and metabolic stability, highlighting the functional integration of microbiota and metabolites under salt stress. These findings support *P. anserina* as a sustainable feed resource and provide a theoretical basis for rational saline soil development via microbiome-informed forage innovation.

## Data Availability

All data generated in this study are included in the main text or supplementary data. The DNA sequencing data results have been deposited in the Figshare repository, with the public access link: https://figshare.com/s/212d64b99076639cfc78. The non-volatile and volatile metabolites data results have been deposited in the Figshare repository, with the public access link: https://figshare.com/s/e1bdd27c6d1101a41ba8.

## References

[B1] Abebe H. Tu Y. (2024). Impact of salt and alkali stress on forage biomass yield, nutritive value, and animal growth performance: a comprehensive review. Grasses 3, 355–368. doi: 10.3390/grasses3040026

[B2] Aranda A. Primo-Catalunya D. Pijuan M. Balcázar J. L. (2024). Draft genome sequence of *Methyloversatilis sp*. Strain NSM2, isolated from a wastewater treatment plant. Microbiol. Res. Announc. 13:e0095224. doi: 10.1128/mra.00952-24 39436067 PMC11556028

[B3] Batt C. A. (2014). “Aflatoxin see mycotoxins: toxicology,” in Encyclopedia of Food Microbiology. Academic Press, 38.

[B4] Campos A. M. Lissi E. Chavez M. Modak B. (2012). Antioxidant activity in heterogeneous and homogeneous system of the resinous exudates from *Heliotropium stenophylum* and *H. sinuatum* and of 3-O-methylgalangin their main component. Boletín Latinoamericano y Del Caribe de Plantas Medicinales y Aromáticas 11, 549–555.

[B5] Cheng J. Zhao L. Liu D. Shen R. Bai D. (2022). *Potentilla anserine L*. polysaccharide protects against cadmium-induced neurotoxicity. Environ. Toxicol. Pharmacol. 90:103816. doi: 10.1016/j.etap.2022.103816 35066145

[B6] Collins M. Nelson C. J. Moore K. J. Barnes R. F. (2017). Forages, Volume 1: An Introduction to Grassland Agriculture. John Wiley and Sons.

[B7] do Prado N. B. de Abreu C. B. Pinho C. S. Junior M. M. de N. Silva M. D. . (2022). Application of multivariate analysis to assess stress by Cd, Pb and Al in basil (Ocimum basilicum L.) using caffeic acid, rosmarinic acid, total phenolics, total flavonoids and total dry mass in response. Food Chem. 367:130682. doi: 10.1016/j.foodchem.2021.130682 34364147

[B8] Du Z. Yang F. Fang J. Yamasaki S. Oya T. Nguluve D. . (2023). Silage preparation and sustainable livestock production of natural woody plant. Front. Plant Sci. 14:1253178. doi: 10.3389/fpls.2023.1253178 37746011 PMC10514673

[B9] Echeverría J. Opazo J. Mendoza L. Urzúa A. Wilkens M. (2017). Structure-activity and lipophilicity relationships of selected antibacterial natural flavones and Flavanones of Chilean Flora. Molecules 22:608. doi: 10.3390/molecules22040608 28394271 PMC6154607

[B10] El Shaer H. M. (2010). Halophytes and salt-tolerant plants as potential forage for ruminants in the Near East region. Small Rumin. Res. 91, 3–12. doi: 10.1016/j.smallrumres.2010.01.010

[B11] Guo P. Chen H. Ma J. Zhang Y. Chen H. Wei T. . (2023). Enzyme-assisted extraction, characterization, and *in vitro* antioxidant activity of polysaccharides from *Potentilla anserina* L. Front. Nutr. 10:1216572. doi: 10.3389/fnut.2023.1216572 37528998 PMC10388540

[B12] Hasanuzzaman M. Nahar K. Brzozowski T. (2022). Plant Stress Physiology: Perspectives in Agriculture. IntechOpen. doi: 10.5772/intechopen.94821

[B13] Hasanuzzaman M. Raihan M. R. H. Masud A. A. C. Rahman K. Nowroz F. Rahman M. . (2021). Regulation of reactive oxygen species and antioxidant defense in plants under salinity. Int. J. Molec. Sci. 22:9326. doi: 10.3390/ijms22179326 34502233 PMC8430727

[B14] Jiao Y. He Q. Li X. Chen Y. Tian T. Cao L. . (2025). Genome-wide identification of starch metabolism gene families in *Potentilla anserina* and the expression pattern in response to abiotic stress factors. BMC Plant Biol. 25:201. doi: 10.1186/s12870-025-06229-y 39953429 PMC11827173

[B15] Keeling L. Tunón H. Olmos Antillón G. Berg C. Jones M. Stuardo L. . (2019). Animal welfare and the united nations sustainable development goals. Front. Vet. Sci. 6:00336. doi: 10.3389/fvets.2019.00336 31649940 PMC6797006

[B16] Li H. Zeng T. Du Z. Dong X. Xin Y. Wu Y. . (2022). Assessment on the fermentation quality and bacterial community of mixed silage of faba bean with forage wheat or oat. Front. Microbiol. 13:519. doi: 10.3389/fmicb.2022.875819 35602069 PMC9114351

[B17] Li X. Wang J. Li S. Yu S. Liu H. Liu Y. (2024). A systematic review on botany, ethnopharmacology, phytochemistry and pharmacology of *Potentilla anserina* L. J. Ethnopharmacol. 333:118481. doi: 10.1016/j.jep.2024.118481 38909825

[B18] Liang S. Liu Y. Y. Lin N. F. (2013). Saline-Alkaline grassland improvement through the bio-engineering technology. Adv. Materials Res. 807–809:1318–1321. doi: 10.4028/www.scientific.net/AMR.807-809.1318

[B19] Liu Y. Cadwallader D. C. Drake M. (2023). Identification of predominant aroma components of dried pea protein concentrates and isolates. Food Chem. 406:134998. doi: 10.1016/j.foodchem.2022.134998 36450193

[B20] Ma Q. Wang H. Wu E. Zhang H. Feng Y. Feng B. (2023). Widely targeted metabolomic analysis revealed the effects of alkaline stress on nonvolatile and volatile metabolites in broomcorn millet grains. Food Res. Int. 171:113066. doi: 10.1016/j.foodres.2023.113066 37330826

[B21] Ma S. Lv L. Meng C. Zhang C. Li Y. (2020). Integrative analysis of the metabolome and transcriptome of sorghum bicolor reveals dynamic changes in flavonoids accumulation under Saline–Alkali Stress. J. Agri. Food Chem. 68, 14781–14789. doi: 10.1021/acs.jafc.0c06249 33274637

[B22] Margaritopoulou T. Kizis D. Kotopoulis D. Papadakis I. E. Anagnostopoulos C. Baira E. . (2022). Corrigendum to: enriched HeK4me3 marks at Pm-0 resistance-related genes prime courgette against Podosphaera xanthii. Plant Physiol. 188, 672–672. doi: 10.1093/plphys/kiab502 34788456 PMC8774742

[B23] Negacz K. Malek Ž. de Vos A. Vellinga P. (2022). Saline soils worldwide: identifying the most promising areas for saline agriculture. J. Arid Environ. 203:104775. doi: 10.1016/j.jaridenv.2022.104775

[B24] Ogunade I. M. Jiang Y. Pech Cervantes A. A. Kim D. H. Oliveira A. S. Vyas D. . (2018). Bacterial diversity and composition of alfalfa silage as analyzed by Illumina MiSeq sequencing: effects of Escherichia coli O157:H7 and silage additives. J. Dairy Sci. 101, 2048–2059. doi: 10.3168/jds.2017-12876 29274960

[B25] Ortner A. Chantrell A. Fay L. B. (2005). Use of 1-nonen-3-one as a flavouring agent (China Patent No. CN1575790A). Available online at: https://patents.google.com/patent/CN1575790A/en

[B26] Parra M. Valenzuela B. Soto S. Modak B. (2016). Antibacterial activity of cuticular components from Heliotropium species, against *Staphylococcus aureus* and *Salmonella tiphymurium*. Boletín Latinoamericano y Del Caribe de Plantas Medicinales y Aromáticas 15, 422–428.

[B27] Pereira M. L. S. Carvalho J. L. V. R. Lima J. M. S. Barbier E. Bernard E. Bezerra J. D. P. . (2022). Richness of Cladosporium in a tropical bat cave with the description of two new species. Mycol. Progress 21, 345–357. doi: 10.1007/s11557-021-01760-2

[B28] Prasad D. S. (2022). Effect of sodic soil and water logged condition on forage crop and their application in breeding. Int. J. Plant Soil Sci. 34, 601–606. doi: 10.9734/ijpss/2022/v34i242679

[B29] Pusztahelyi T. Holb I. J. Pócsi I. (2015). Secondary metabolites in fungus-plant interactions. Front. Plant Sci. 6:00573 doi: 10.3389/fpls.2015.00573 26300892 PMC4527079

[B30] Robinson P. H. Grattan S. R. Getachew G. Grieve C. M. Poss J. A. Suarez D. L. (2004), Biomass accumulation potential nutritive value of some forages irrigated with saline-sodic drainage water. Animal Feed Sci. Technol. 111, 175–189. doi: 10.1016/S0377-8401(03)00213-X.

[B31] Rodríguez Martín-Aragón V. Trigal Martínez M. Cuadrado C. Daranas A. H. Fernández Medarde A. Sánchez López J. M. (2023). OSMAC approach and cocultivation for the induction of secondary metabolism of the *Fungus Pleotrichocladium opacum*. ACS Omega 8, 39873–39885. doi: 10.1021/acsomega.3c06299 37901491 PMC10601420

[B32] Schubert K. Groenewald J. Z. Braun U. Dijksterhuis J. Starink M. Hill C. F. . (2007). Biodiversity in the *Cladosporium herbarum complex (Davidiellaceae, Capnodiales)*, with standardisation of methods for Cladosporium taxonomy and diagnostics. Stud. Mycol. 58, 105–156. doi: 10.3114/sim.2007.58.05 18490998 PMC2104742

[B33] Serrapica F. Uzun P. Masucci F. Napolitano F. Braghieri A. Genovese A. . (2020). Hay or silage? How the forage preservation method changes the volatile compounds and sensory properties of Caciocavallo cheese. J. Dairy Sci. 103, 1391–1403. doi: 10.3168/jds.2019-17155 31785866

[B34] Sharma A. Shahzad B. Rehman A. Bhardwaj R. Landi M. Zheng B. (2019). Response of phenylpropanoid pathway and the role of polyphenols in plants under abiotic stress. Molecules 24:2452. doi: 10.3390/molecules24132452 31277395 PMC6651195

[B35] Sutthiwong N. Lekavat S. Dufossé L. (2023). Involvement of versatile bacteria belonging to the genus arthrobacter in milk and dairy products. Foods 12:1270. doi: 10.3390/foods12061270 36981196 PMC10048301

[B36] Thiex N. Novotny L. Crawford A. (2012). Determination of ash in animal feed: AOAC official method 942.05 revisited. J. AOAC Int. 95, 1392–1397. doi: 10.5740/jaoacint.12-129 23175971

[B37] Wang J. Zhang J. Zhao B. Wang X. Wu Y. Yao J. (2010). A comparison study on microwave-assisted extraction of *Potentilla anserina* L. polysaccharides with conventional method: molecule weight and antioxidant activities evaluation. Carbohydr. Polym. 80, 84–93. doi: 10.1016/j.carbpol.2009.10.073 19751758

[B38] Wang T. Zhang J. Shi W. Sun J. Xia T. Huang F. . (2022). Dynamic changes in fermentation quality and structure and function of the microbiome during mixed silage of *Sesbania cannabina* and *Sweet Sorghum* grown on Saline-Alkaline Land. Microbiol. Spectr. 10, e02483–e02422. doi: 10.1128/spectrum.02483-22 36190422 PMC9604195

[B39] Wu Y. H. Wang H. Liu M. Li B. Chen X. Ma Y. T. . (2021). Effects of native *Arbuscular Mycorrhizae* isolated on root biomass and secondary metabolites of *Salvia miltiorrhiza Bge*. Front. Plant Sci. 12:617892. doi: 10.3389/fpls.2021.617892 33603763 PMC7884620

[B40] Xian F. Yang L. Ye H. Xu J. Yue X. Wang X. (2024). Revealing the mechanism of aroma production driven by high salt stress in *Trichomonascus ciferrii* WLW. Foods 13:1593. doi: 10.3390/foods13111593 38890822 PMC11172348

[B41] Xu L. He J. Meng Y. Zheng Y. Lu B. Zhang J. . (2024). Enhancing drought resistance in *Pinus tabuliformis* seedlings through root symbiotic fungi inoculation. Front. Plant Sci. 15:1446437. doi: 10.3389/fpls.2024.1446437 39228833 PMC11368727

[B42] Yang K. Su W. Mu Y. Liu X. Liu Y. Lu Y. (2025). Microbial absolute quantification and metabolomics reveal the impact of partial KCl substitution for NaCl on the flavor quality of Zao Chili. Food Biosci. 68:106495. doi: 10.1016/j.fbio.2025.106495

[B43] Zeng L. Watanabe N. Yang Z. (2019). Understanding the biosyntheses and stress response mechanisms of aroma compounds in tea (Camellia sinensis) to safely and effectively improve tea aroma. Crit. Rev. Food Sci. Nutr. 59, 2321–2334. doi: 10.1080/10408398.2018.1506907 30277806

[B44] Zhang C. Zhou D. F. Wang M. Y. Song Y. Z. Zhang C. Zhang M. M. . (2024). *Phosphoribosylpyrophosphate synthetase* as a metabolic valve advances *Methylobacterium/Methylorubrum phyllosphere* colonization and plant growth. Nat. Commun. 15:5969. doi: 10.1038/s41467-024-50342-9 39013920 PMC11252147

[B45] Zhang K. Zhang T. T. Guo R. R. Ye Q. Zhao H. L. Huang X. H. (2023). The regulation of key flavor of traditional fermented food by microbial metabolism: a review. Food Chem. 19:100871. doi: 10.1016/j.fochx.2023.100871 37780239 PMC10534219

[B46] Zhihao D. Junfeng L. Siran W. Dong D. Tao S. (2022). Time of day for harvest affects the fermentation parameters, bacterial community, and metabolic characteristics of sorghum-sudangrass hybrid silage. mSphere 7:e00168–e00122. doi: 10.1128/msphere.00168-22 35862805 PMC9429962

[B47] Zhou B. Chen Y. Zeng L. Cui Y. Li J. Tang H. . (2022). Soil nutrient deficiency decreases the postharvest quality-related metabolite contents of tea (Camellia sinensis (L.) Kuntze) leaves. Food Chem. 377:132003. doi: 10.1016/j.foodchem.2021.132003 35008025

[B48] Zhu Q. Chen L. Peng Z. Zhang Q. Huang W. Yang F. . (2022). Analysis of environmental driving factors on core functional community during Daqu fermentation. Food Res. Int. 157:111286. doi: 10.1016/j.foodres.2022.111286 35761594

